# Ultrasound Screening of Temporomandibular Joint Pathology in Children with Chronic Kidney Disease

**DOI:** 10.3390/dj13020084

**Published:** 2025-02-15

**Authors:** Natalia Sergeevna Morozova, Alina Alekseevna Elovskaya, Ekaterina Andreevna Maslikova, Oleg Ivanovich Admakin, Arif Fuad Allahverdiyev, Ellina Valerievna Velichko, Larisa Dmitrievna Maltseva, Pavel Pavlovich Tregub, Olga Leonidovna Morozova

**Affiliations:** 1 Department of Dental Diseases Propaedeutics, E.V. Borovsky Institute of Dentistry, I.M. Sechenov First Moscow State Medical University (Sechenov University), 117418 Moscow, Russia; 2 Department of Preventive Dentistry and Orthodontics, E.V. Borovsky Institute of Dentistry, I.M. Sechenov First Moscow State Medical University (Sechenov University), 121059 Moscow, Russia; 3 Department for Therapeutic Dentistry Propaedeutics, A.l. Evdokimov Institute of Dentistry, 127006 Moscow, Russia; 4 Department of Pathophysiology, Institute of Digital Biodesign and Modeling of Living System, I.M. Sechenov First Moscow State Medical University (Sechenov University), 13-1 Nikitsky Boulevard, 119019 Moscow, Russia

**Keywords:** children, chronic kidney disease (CKD), hemodialysis, temporomandibular joint (TMJ), TMJ pathology, ultrasonography

## Abstract

**Background/Objectives**: Chronic kidney disease (CKD) influences different organs including the temporomandibular joint (TMJ). This study aims to identify structural and functional TMJ changes in children with CKD using ultrasound as the least invasive and most accessible method. **Methods**: TMJ changes were examined using ultrasound screening in 40 children. The first group (control, *n* = 10) included children with normal occlusion without TMJ complaints. The second group (*n* = 10) included children with CKD stage 1 and 2. The third group (*n* = 10) included patients on hemodialysis after renal transplantation. The forth group (*n* = 10) included patients at least 6 months after renal transplantation. **Results**: The size of the anterior section of the right TMJ gap in the third group was the largest among all the groups studied (1.085 mm) and statistically significantly different from the first group (0.570 mm; *p* = 0.001) and the second one (0.665 mm, *p* = 0.001). The width of the middle section was also greatest in the third group and statistically significantly different when compared to the first and second groups (0.390 mm; *p* = 0.023 and 0.340 mm; *p* < 0.001, respectively). A posterior articular gap width differences between the individual patient groups under study were not statistically significant in a posteriori comparison with Bonferroni correction. Statistical significance of differences between all groups when comparing the gap width was found in all sections of the left TMJ. The frequency of anterior disc displacement between groups ranged from 50 to 100% in all groups studied and was not statistically different when comparing right and left TMJs between groups (*p* = 0.084 and *p* = 0.662, respectively). **Conclusions**: CKD children have different TMJ changes, so TMJ ultrasound could screen joint pathology at early stages, and dental specialists can start timely rehabilitation.

## 1. Introduction

Chronic kidney disease (CKD) is a supra-nosological concept characterized by a persistent decline in renal function for three months or more, regardless of the nosological diagnosis, leading to the replacement of normal anatomical structures by fibrosis [1].

Changes in the structure of the hard tissues of the maxillofacial region as a result of CKD occur due to a systemic metabolic disorder and are united by the term ‘Mineral and bone disorders associated with chronic kidney disease’ [2,3,4,5,6].

Phosphorus is a key element in the disturbance of mineral homeostasis [7,8]. Due to impaired metabolism of phosphorus, blood calcium levels also decrease [9,10], and hypocalcaemia induces parathyroid hormone secretion and secondary hyperparathyroidism, which leads to a quantitative increase in active osteoclasts in bone and, consequently, the development of renal osteodystrophy [10,11].

Growth impairment or stunting is also a frequent comorbidity of CKD in children [12] due to the insensitivity of bone tissue to growth hormone [13] and reduced levels of circulating insulin-like growth factor [14,15]. The situation is complicated by corticosteroid drug intake during underlying disease treatment, the side effect of which is stimulation of somatostatin release, which in turn suppresses growth hormone release by the pituitary gland [16].

As a result of renal tissue damage, calcification of tissues (cartilaginous and connective tissues, vessels), immature bone matrix formation, and unstable cortical bone structure are observed, which becomes quite fragile and rapidly atrophies with replacement by less durable trabecular bone tissue, making joint surfaces unstable against loads [17].

The above-mentioned processes can both directly and indirectly affect the structures of the temporomandibular joint (TMJ) [18,19]. To determine them in children with CKD, it is necessary to select the most rational method of diagnosis.

One such method is ultrasonography (USG). This method of investigation is non-invasive, easily reproducible, cost-effective, affordable in terms of equipment and price, and most importantly, it allows for dynamic assessment without ionizing radiation [20,21,22]. The possibility of simultaneous USG of the kidneys and TMJ in children with these pathologies is also an undeniable advantage. For this reason, USG is the method of choice in the pediatric age group as a screening tool to evaluate the TMJ complex.

USG is a method based on the reflection of sound waves from different density structures, which differ in the intensity of reflection of ultrasound waves (in our case, 12 mHz—high-sensitivity ultrasound) [20,23,24].

Using the proposed method, it is possible to obtain data on the anatomical and morphological features of TMJ structure and development. For example, it is possible to diagnose the presence/absence of articular disc dislocation (hypoechogenic formation); to evaluate disc mobility when opening and closing the mouth as well as the integrity of the articular capsule disc (isoechogenic structure); to visualize the articular surfaces of the condyle and temporal bone (hyperechogenic formations); and to determine the size and nature of the surfaces of these structures, as well as the upper and lower floors of the joint. In addition to the above, ultrasound can be used to assess the increase in the level of synovial fluid in the joint cavity by determining the parameters of the inferior articular gap [25,26].

The following TMJ pathologies can be diagnosed by ultrasound analysis: arthritis, arthrosis, ankylosis, the formation of hydroxyapatite crystals in the joint structures (calcification), osteodystrophy of the cortical plates of the articular surfaces, the presence of joint effusion (traumatic, infectious, dystrophic synovitis), and anterior/posterior displacement of the articular disc [25,26,27].

The relevance of our study is confirmed by the fact that the ultrasound diagnostics of TMJ pathologies in children with CKD with mineral and bone disorders is insufficient in the explored scientific literature. In addition, the rationality of using this method of research seems obvious to us, since patients routinely undergo renal ultrasonic diagnostics; therefore, there is an opportunity to conduct a one-stage dynamic examination of TMJ structures, avoiding additional financial expenses and excluding increased radiation and ionizing load.

Thus, our study aim is to evaluate the effectiveness of ultrasound for screening temporomandibular joint pathology in children with chronic kidney disease.

## 2. Materials and Methods

### 2.1. Study Design

This pilot prospective one-stage multicenter diagnostic study was aimed at determining the possibility of detecting changes in TMJ structures in children with CKD using ultrasound examination as part of dynamic follow-up.

The study was carried out in the period from February to May 2022 on the basis of the following clinical centres: E. V. Borovsky Institute of Dentistry of Sechenov University; the nephrology department of Moscow Children’s Hospital № 9, named after G.N. Speransky; and surgical department № 1 of the National Medical Centre of Transplantology and Artificial Organs, named after Academician V.I. Shumakov of the Ministry of Health of Russia.

Patients were selected by the method of continuous solid observation. The study was a pilot study, so no preliminary calculation of the sample size was performed.

The criteria for inclusion in the study were a diagnosis of CKD and informed voluntary consent of the patient or their legal representative; only those patients who had completely passed the stage of TMJ diagnosis by USG and had not previously undergone orthodontic treatment were included in the study. Exclusion criteria were the presence of concomitant general medical pathology other than CKD, including endocrine diseases affecting bone metabolism, previous orthodontic treatment, and patients‘ or their legal representatives’ refusal to participate in any study stages. The first group (control group) consisted of healthy patients with normal occlusion without TMJ complaints. The second group included patients with CKD stage 1 and 2. The third group included patients undergoing hemodialysis after renal transplantation. The fourth group included patients at least 6 months after renal transplantation.

Ultrasound was performed in all patients using a Samsung SONO ACE R3 device (LN5 sensor—12 MHz in longitudinal and transverse scanning modes).

**Figure 1:**
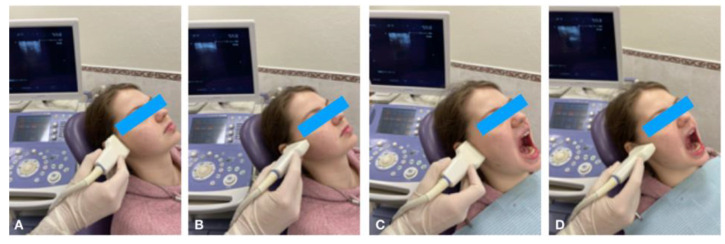
Ultrasonography of TMJ in longitudinal (**A**) and transverse (**B**) scans in the closed mouth position; longitudinal (**C**) and transverse (**D**) scans in the open mouth position.

USG of the TMJ was used to assess the articular disc structure of the right and left joints, as well as the presence or absence of its mobility to the anterior, posterior, and lateral sides. Ultrasound control was performed in the patient’s semi-recumbent position. By using a linear transducer placed in the projection of the articular surface of the mandibular head, a clear contour of the position and structure of the articular disc was visualized. Oblique and transverse scans were used. During the present study, the patient performed opening and closing movements of the mandible, and the degree of movement of the articular disc was determined (Figure 1). The following TMJ parameters were determined during USG: the contours and position of the articular disc, its echogenicity and degree of homogeneity, the degree of its mobility relative to the mandibular head, the height of the articular disc in the anterior, middle, and posterior parts, and the height of the lower articular gap. At the correct position of the high-frequency transducer, the contour of the mandibular head was visualized as a thin hyperechogenic continuous line. Further, the lower articular gap was visualized in the form of a uniform anechogenic stripe up to 2 mm in height. Above the head and the lower articular gap, a hypoechogenic, soft-tissue, homogeneous, mobile formation with a fine-grained structure in the form of a double-curved lens—the articular disc—was located. The anterior and posterior thickenings were distinguished in it, as well as the middle narrowed part (normal 1–2 mm). Movement of the articular disc was determined by opening and closing of the mouth, which in the norm reached 14–15 mm. Movement in the norm occurred without deviation of the mandible and without articular noise. In cases of incomplete movement of the articular disc, the presence of partial repositioning of the articular disc was noted, and in cases of impossibility of its movement, a complete absence of repositioning was noted. In anterior dislocation, the articular disc was displaced anteriorly, and its deformation occurred, with an increase in anterior dimensions and decrease in posterior thickening. This articular deformation led to a change in the disc structure to an echogenic, layered, heterogeneous structure with disturbed fine dispersion.

### 2.2. Ethical Review

All stages of the study were approved by the Local Ethical Committee of the Federal State Autonomous Educational Institution of Higher Education I.M. Sechenov First Moscow State Medical University of the Ministry of Health of the Russian Federation (Sechenov University) (protocol No. 01-22 of 20.01.2022).

### 2.3. Statistical Analysis

The data obtained during the study were entered into Microsoft Excel 2010 spreadsheets and coded for further statistical processing.

The distribution of quantitative characteristics (joint gap size) was assessed using the Shapiro–Wilk test. Because, when comparing each of the characteristics in at least one of the comparison groups, the distribution did not conform to the normal distribution, the nonparametric Kruskall–Wallis H-criterion was used to test the hypothesis that there were differences between all groups at the level of the general population. In case of acceptance of the alternative hypothesis about the presence of differences, a posteriori pairwise comparison of the sign in the groups was carried out using Dunn’s criterion with Bonferroni correction.

For comparison of qualitative features (presence of anterior disc displacement) between groups, conjugation tables and calculation of Fisher’s exact criterion were applied due to the presence of values less than 5 in individual cells.

Median (Me) and interquartile range (QR) values were used to describe quantitative features (joint gap width). For qualitative features (presence of anterior disc displacement), absolute value (n), specific gravity (%), and 95% confidence interval (95% CI) values were used.

Statistical analysis of the data was performed using SPSS 24.0 software (SPSS Inc., Chicago, IL, USA).

## 3. Results

After applying inclusion and exclusion criteria, the present study examined the dimensions of the articular gaps of the left and right TMJ in 40 patients, of whom 16 (40%) were male and 24 (60%) were female. The mean age of the patients was 13.8 ± 2.6 years (9 to 18 years). Characteristics of the groups and patients included in the study are presented in Table 1.

Comparison of the dimensions of all the articular gap sections in the right TMJ revealed the presence of statistically significant differences between all the study groups in each of the sections except for the inferior gap (Table 2).

As shown in Table 2, the size of the anterior aspect of the right TMJ gap in the hemodialysis patient group was the largest among all the groups studied (1.085 mm) and statistically significantly different from the groups of patients who did not have CKD (0.570 mm, *p* = 0.001) and those who had CKD grade 1–2 (0.665 mm, *p* = 0.001).

The width of the middle section of the articular cleft of the right TMJ was also greatest in the group of patients undergoing hemodialysis and was statistically significantly different when compared to the groups of patients who did not have CKD (0.390 mm) and who had grade 1–2 CKD (0.340 mm; *p* = 0.023 and *p* < 0.001, respectively). The size of the mid-articular gap also differed when comparing the groups of patients who had grade 1–2 CKD (0.340 mm) and those who had undergone kidney transplantation (0.815 mm, *p* = 0.003).

Despite the differences found when comparing all groups, a posterior articular gap width differences between the individual patient groups under study were not statistically significant in a posteriori comparison with Bonferroni correction.

In turn, statistical significance of differences between all groups when comparing the gap width was found in all sections of the left TMJ (Table 3).

The width of the anterior aspect of the left TMJ gap was more than 1.5 times greater in the group of renal transplant patients than in patients without CKD (0.830 mm and 0.510 mm, respectively; *p* = 0.004).

The median dimensions of the middle section of the left TMJ slit were 0.785 mm in the hemodialysis and transplant patient groups and were statistically significantly different compared to the group of patients who had no CKD (0.310 mm; *p* = 0.003 and *p* = 0.001, respectively).

The posterior articular slit width of the left TMJ was also greatest in the hemodialysis patient groups (0.850 mm). Pairwise comparisons revealed statistically significant differences when comparing this group with the groups of patients who had no CKD (0.440 mm) and those who had grade 1–2 CKD (0.470 mm; *p* = 0.025 for both comparisons). Statistically significant differences were also found when comparing the posterior gap width of the left TMJ in the above two groups with the group of patients who had undergone kidney transplantation (0.770 mm; *p* = 0.029 for both comparisons).

The greatest width of the left TMJ inferior gap was found in the group of patients with grade 1–2 CKD (0.025 mm). Although all other groups had a median inferior gap size of 0.100 mm, statistically significant differences were found only when comparing the group of patients with grade 1–2 CKD with the groups of patients on hemodialysis and those who had undergone renal transplantation (*p* = 0.040 for both comparisons).

**Figure 2:**
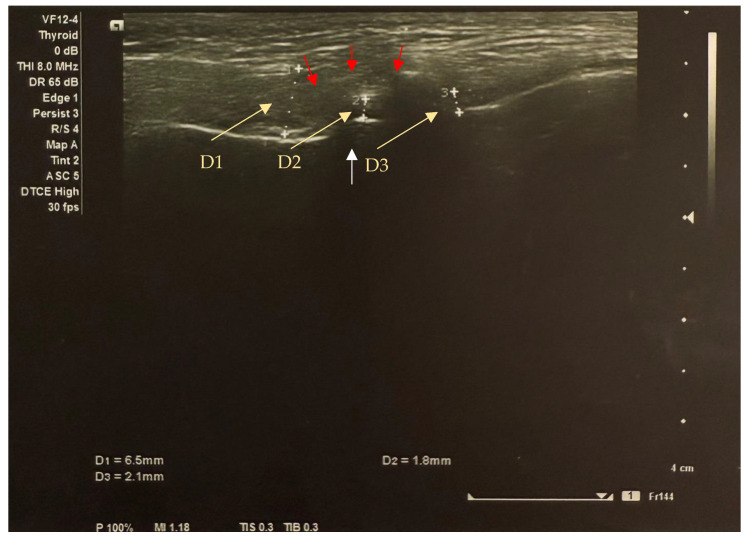
Transverse image of the left TMJ ultrasound in the closed mouth position shows anterior disc displacement (yellow arrows indicate the distance between disc thickness markers): anterior section (D1) = 6.5 mm; middle section (D2) = 1.8 mm; posterior section (D3) = 2.1mm. The white arrow shows the condyle (the condyle surface is hyperechogenic (high reflection of sound waves) and appears white on ultrasound images); the red arrows show the sutural capsule (the connective tissues represented by the joint capsule are isoechogenic (intermediate reflection of sound waves) and appear inhomogeneously grey on ultrasound images).

The frequency of anterior disc displacement (Figure 2) between groups ranged from 50 to 100% in all groups studied and was not statistically different when comparing both (right and left) TMJs between groups (*p* = 0.084 and *p* = 0.662, respectively) (Table 4).

## 4. Discussion

This study was conducted as part of dissertation research and a primary assessment of the maxillofacial region and analysis of the prevalence of these pathological conditions in pediatric patients at different stages of CKD. Nowadays, there is a tendency to develop pathologies of the TMJ at an early age and in children without generalized pathologies, which disrupts normal craniofacial growth and development.

Today, there are enough methods for imaging TMJ structures in pediatric practice, the main ones being magnetic resonance imaging (MRI), cone beam computed tomography (CBCT), and USG [27]. Currently, MRI is the gold standard for the diagnosis of TMJ pathological conditions due to its high level of soft tissue contrast [28]. At the same time, the main disadvantages of MRI are its inaccessibility in some public medical centres, high cost, and limited use due to many contraindications (claustrophobia, ferromagnetic metal implants, etc.) [29]. CBCT can be used to assess the shape, position, and cortication of the morphological structures of the TMJ complex, but it is unable to visualize the soft tissue components of the TMJ and cannot be used in children for dynamic monitoring of the joint due to the high radiation load [30].

Considering the development of bone and mineral disorders in most patients with CKD, the use of ultrasound as a primary screening method of TMJ condition allows us to identify subclinical forms of TMJ pathological conditions even before the appearance of clinical symptoms. According to the literature, it has been observed that most TMJ pathologies are asymptomatic. When searching the relevant literature, it was found that the benefits of USG in the diagnosis of TMJ pathologies in children with CKD had not been previously described. However, the works of other authors on ultrasound diagnosis of juvenile idiopathic arthritis (a disease that occurs in children up to 18 years of age) were taken to confirm the possibility of visualizing TMJ changes in CKD, in which the possibility of determining such characteristics as the width of the anterior, middle, and posterior parts of the articular disc, as well as the width of the articular gap, was indicated [23,31,32,33]. Since no data reflecting normal values of disc thickness on ultrasound in the pediatric population were found in the literature, the control group was taken as the gold standard. At the same time, in the scientific works of some authors who conducted studies of TMJ pathology in pediatric patients using ultrasound, there are data that were proposed as guidelines for specialists: it is suggested to take the threshold value of the joint capsule width of 1.2 mm as the norm; if this value increases more than 1.2 mm, one should consider the obtained values as a sign of pathological processes (synovitis and the presence of effusion in the joint), which requires clarification using more accurate imaging methods [32,34,35]. Together with these characteristics, the contour of the articular surfaces was also assessed, and as a result of analyzing all the above parameters of the articular surfaces, articular disc, and articular chamber, conclusions were drawn about the presence of such diseases as secondary osteoarthritis and anterior displacement of the articular disc as the main TMJ disorders in CKD.

A group of researchers conducted a comparative evaluation of the TMJ using ultrasound and CBCT data, which showed a false-positive rate of 33% for ultrasound diagnosis in the presence of anomalies, especially of the mandibular head [22]. However, according to the same authors, the positive predictive value of ultrasound for the detection of normal contours of the cortical plate of the condyle was noted as 61%. From this, the researchers concluded that when ultrasound shows a normal contour, there is a moderate probability that this result is accurate. A negative predictive value of 67% was found, highlighting the positive aspect of using ultrasound to rule out abnormalities when a normal result is obtained: a negative result when ultrasound is used is a good predictor of the absence of disease. At the same time, when abnormalities are detected on ultrasound, changes in the different structures of the joint are necessarily detected on CBCT. Thus, the authors summarized that the use of TMJ ultrasound is well suited for initial screening and exclusion of pathologies, but due to its variable sensitivity and specificity, caution should be exercised when interpreting the data and, if there is evidence of pathology, it should be supplemented with high-resolution imaging methods such as CBCT [22].

High-resolution ultrasound, defined as ultrasound resolution of 12 MHz or more, was used in this study because it improves sensitivity and specificity in detecting TMJ lesions compared to low-resolution ultrasound, defined as ultrasound resolution <12 MHz [20,23]. Also, a study by Jank et al. [24] showed that high-resolution ultrasound can detect TMJ pathology at early stages even before the onset of clinical symptoms, which is particularly relevant in pediatric practice. As advances in ultrasound probe technology continue, in the future, the use of a higher-frequency ultrasound probe (18 MHz) will allow for more detailed tissue differentiation will improve diagnostic accuracy [36].

To date, MRI is the gold standard for imaging soft tissue structures of the TMJ [37], namely the articular disc, assessing its size, integrity, and position. There are many studies in the literature that have comparatively assessed the diagnostic accuracy of TMJ disorders. However, although MRI is the gold standard, this study cannot be performed dynamically in real time, which is one of its limitations. We also analyzed works on the diagnosis and comparison of diagnostic methods (MRI, USG) of TMJ pathologies in children and concluded that although MRI has the highest sensitivity and specificity, USG allows dynamic monitoring, which significantly increases its diagnostic value [23,27,33]. Various studies report 83–95% [38,39] and 62.5–71.4% [37] accuracy of ultrasound in assessing the position of the articular disc. In normality, the anterior and posterior widths of the disc are equal, with the middle portion being smaller in size compared to its anterior and posterior portion; this is due to the normal anatomy of the articular disc (bilaterally curved lens). These articular disc thickness dimensions allow us to estimate the size of the joint spaces and suggest the position of the condyle in the articular fossa [35,36]. All groups of patients except the control group showed an increase in the anterior articular disc thickness compared to the posterior one to different degrees (of the joint capsule in the anterior region), which confirms the tendency for anterior displacement of the articular disc in the sagittal plane in the studied groups. In this case, the group of patients undergoing hemodialysis among all studied groups showed a greater tendency and was more statistically significantly different from patients without CKD pathology. Moreover, different widths of the articular disc of the right and left TMJs were observed, which suggests asymmetry of the disc position and, as a consequence, asymmetry of the position of the mandibular heads. These intra-articular changes indicate the initial stages of joint dysfunction and, consequently, abnormal growth and development of the facial skeleton, requiring correction in the early stages of development.

## 5. Conclusions

This study showed that children with CKD tend to develop pathology of TMJ structures. Our proposed ultrasound method is of high diagnostic value in this category of patients but has several limitations because the assessment of the position of the articular disc is possible only in the sagittal plane. In children on hemodialysis and after renal transplantation, the most pronounced statistically significant changes were found in all parts of the left TMJ and in the anterior and middle parts of the right TMJ. Although ultrasound alone cannot provide a comprehensive assessment of complex degenerative changes, based on the results obtained by ultrasound of the TMJ in children with CKD, even in the absence of clinical symptoms, when pathology is detected in them, it is justification for the use of CBCT as a definitive diagnostic standard. Thus, the integration of TMJ ultrasound into the mandatory protocol of examination of children with CKD will make it possible to screen joint pathology at early stages and refer such patients to dental specialists for timely rehabilitation.

## 6. Limitations

The sample size was limited to the number of patients admitted to the local hospital during the study period, allowing for a single point of observation. Therefore, this is a pilot study. In particular, the analysis of the left/right TMJ anterior disc displacement index did not reveal statistically significant differences due to the insufficient power of the study. To confirm the findings, it is necessary to conduct longitudinal studies on large samples using probabilistic selection of observation units. We should not ignore the value of suspicious ultrasound signs, as it is a non-invasive relevant approach to diagnose and monitor TMJ pathology in children with CKD.

Ultrasound diagnosis has no limitation in frequency and can be performed any number of times. But the limitation of image clarity is due to the area of the transducer and the technical characteristics of the ultrasound machine. In addition, ultrasound has less resolution than cone beam computed tomography. The qualification and experience of the specialist performing the study play an important role.

## Figures and Tables

**Table 1:** Characteristics of the sample of patients.

Patient groups Characteristics	Control	CKD Grade 1–2	Hemodialysis	After Renal Transplantation
**n (number of patients)**	10	10	10	10
**Age, years (M ± SD)**	14.2 ± 1.2	13.6 ± 2.0	12.6 ± 3.0	14.8 ± 3.4
**Gender (female), n (%)**	8 (80%)	6 (60%)	3 (30%)	7 (70%)

Note: CKD—chronic kidney disease.

**Table 2:** Width of the articular gap of the right TMJ in the compared groups of patients.

TMJ Section	Patient groups				
	Control, Me (QR)	CKD Grade 1–2, Me (QR)	Hemodialysis, Me (QR)	After Renal Transplantation, Me (QR)	Significance of the H-Criterion, *p*
**Anterior section, mm**	0.570 (0.470–0.680)	0.665 (0.460–0.690)	1.085 ***, ## (0.903–1.190)	0.755 (0.720–0.810)	<0.001
**Middle section, mm**	0.390 (0.360–0.560)	0.340 (0.300–0.490)	0.910 *, ### (0.740–1.080)	0.815 ## (0.570–0.910)	<0.001
**Posterior section, mm**	0.530 (0.460–0.60)	0.435 (0.400–0.470)	0.815 (0.097–1.08)	0.350 (0.230–0.450)	0.035
**Inferior gap, mm**	0.100 (0.100–0.100)	0.155 (0.100–0.250)	0.100 (0.100–0.100)	0.100 (0.100–0.100)	0.077

Note: Me (QR)—median (Me) and interquartile range (QR) values; CKD—chronic kidney disease; *—significant differences in pairwise comparison with the group of patients from the control group, taking into account the Bonferroni correction; #—significant differences in pairwise comparison with the group of patients who had 1–2 grade CKD, taking into account the Bonferroni correction; *p* < 0.05 (*, correspondingly); *p* < 0.01 (##, correspondingly); *p* < 0.001 (***, ###, correspondingly).

**Table 3:** Width of the articular gap of the left TMJ in the compared groups of patients.

TMJ Section	Patient Groups				
	Control, Me (QR)	CKD Grade 1-2, Me (QR)	Hemodialysis, Me (QR)	After Renal Transplantation, Me (QR)	Significance of the H-Criterion, *p*
**Anterior section, mm**	0.510 (0.450–0.620)	0.660 (0.490–0.70)	0.750 (0.410–1.170)	0.830 ** (0.750–0.890)	0.006
**Middle section, mm**	0.310 (0.280–0.470)	0.435 (0.340–0.530)	0.785 *** (0.650–1.020)	0.785 *** (0.650–0.960)	<0.001
**Posterior section, mm**	0.440 (0.370–0.620)	0.470 (0.400–0.540)	0.850 **, ## (0.710–1.210)	0.770 *, # (0.640–0.850)	0.001
**Inferior gap, mm**	0.100 (0.100–0.210)	0.205 (0.170–0.300)	0.100 # (0.100–0.200)	0.100 # (0.100–0.200)	0.015

Note: Me (QR)—median (Me) and interquartile range (QR) values; CKD—chronic kidney disease; *—significant differences in pairwise comparison with the group of patients from the control group, taking into account the Bonferroni correction; #—significant differences in pairwise comparison with the group of patients who had 1–2 grade CKD, taking into account the Bonferroni correction; *p* < 0.05 (*, #, correspondingly); *p* < 0.01 (**, ##, correspondingly); *p* < 0.001 (***, correspondingly).

**Table 4:** Frequency of anterior disc displacement in the right and left TMJ in the compared patient groups.

TMJ Section	Patient groups				
	Control, n, % (95% CI)	CKD grade 1–2, n, % (95% CI)	Hemodialysis, n, % (95% CI)	After Renal Transplantation, n, % (95% CI)	Significance of Fisher’s Exact Criterion, *p*
**Anterior disc displacement in the right TMJ**	5, 50.0% (22.4–77.6%)	7, 70.0% (39.4–90.7%)	8, 80.0% (49.7–95.6%)	10, 100% (100.0–100.0%)	0.084
**Anterior disc displacement in the left TMJ**	6, 60.0% (30.4–84.7%)	8, 80.0% (49.7–95.6%)	6, 60.0% (30.4–84.7%)	5, 50.0% (22.4–77.6%)	0.662

Note: CI—confidence interval; CKD—chronic kidney disease; TMJ—temporomandibular joint.

## Data Availability

Data are unavailable due to privacy or ethical restrictions.

## Abbreviations

The following abbreviations are used in this manuscript:

CKD	chronic kidney disease
TMJ	temporomandibular joint
USG	ultrasonography
Me	median
QR	interquartile range
CI	confidence interval
MRI	magnetic resonance imaging
CBCT	cone beam computed tomography
